# Improving Output Efficiency of InGaN-Based MQW Green Laser Diodes by Modulating Indium Content of Quantum Barriers and Using Composite Lower Waveguide Layers

**DOI:** 10.3390/nano12152581

**Published:** 2022-07-27

**Authors:** Zhenyu Chen, Feng Liang, Degang Zhao, Jing Yang, Ping Chen, Desheng Jiang

**Affiliations:** 1 State Key Laboratory of Integrated Optoelectronics, Institute of Semiconductors, Chinese Academy of Sciences, Beijing 100083, China; chenzhenyu@semi.ac.cn (Z.C.); yangjing333@semi.ac.cn (J.Y.); pchen@semi.ac.cn (P.C.); dsjiang@red.semi.ac.cn (D.J.); 2 College of Materials Science and Opto-Electronic Technology, University of Chinese Academy of Sciences, Beijing 100049, China; 3 Center of Materials Science and Optoelectronics Engineering, University of Chinese Academy of Sciences, Beijing 100049, China

**Keywords:** InGaN MQW, carrier recombination, green laser diodes, InGaN waveguide, quantum barrier

## Abstract

Potential barriers between the waveguide layer and MQW active region may influence injection efficiency significantly, which is important in improving output characteristics of GaN-based green laser diodes (LDs). In this study, potential barriers and injection efficiency of LDs are investigated by simulation methods. It is found that different indium content in quantum barrier layers results in different potential barrier heights, leading to different recombination rates in upper and lower waveguide layers, and the injection efficiency can be modulated effectively. An eclectic choice of indium content can suppress recombination in two waveguide layers, improving the output characteristics of green LDs. Additionally, a composite lower waveguide layer structure is proposed to reduce the negative effect of potential barriers. High output power and low threshold current are achieved owing to the reduction in electron injection blockage and hole leakage effects.

## 1. Introduction

InGaN-based green laser diodes (LDs) have attracted great attention for many years and many studies have been made due to their significant application in diverse fields, such as laser display, laser printers, and biological sensing [1,2,3,4]. When fabricating high-performance green LDs, both optical and electrical performances of LDs should be taken into consideration [5,6,7]. Compared to InGaN-based blue LDs, which are relatively mature [3], InGaN-based green LDs are still facing many important technical problems [7]. Firstly, the effective refractive index contrast between the waveguide layer and cladding layer will be reduced with the increase in lasing wavelength, leading to insufficient optical confinement [8,9,10]. Thus, green LDs will suffer from an additional optical loss which will decrease the total efficiency. Carrier injection efficiency is another significant technical problem that can greatly influence the threshold current density and slope efficiency of LDs [11,12]. It has been reported that holes can overflow from multiple quantum wells to the lower waveguide layer, inducing severe carrier recombination in waveguides [13,14]. Many studies have investigated electron leakage to the upper waveguide layer and p-cladding layer [15,16]. Those additional electrical losses will deteriorate the electrical performance of green LDs.

However, current reports on the purpose of optimizing carrier distribution focused mainly on the electron leakage to the p-side and hole overflow to the n-side. Additionally, injection efficiency in InGaN-based green LDs has not been studied thoroughly, although this issue is important to green LD’s fabrication. In this article, carrier blocking effects, carrier leakage, and conflicts between carrier injection efficiency and optical confinement in green InGaN-based LDs are studied by using the two-dimensional simulator LASTIP (Crosslight Software Inc., Burnaby, BC, Canada). The influence of indium content in quantum barriers on green LDs is analyzed in detail. Furthermore, a new structure with a composite lower waveguide layer is proposed in order to increase carrier injection efficiency with little loss in an optical confinement. The green LDs with this new waveguide structure have shown significant improvement in both threshold current and slope efficiency.

## 2. Device Structures and Methods

**Figure 1:**
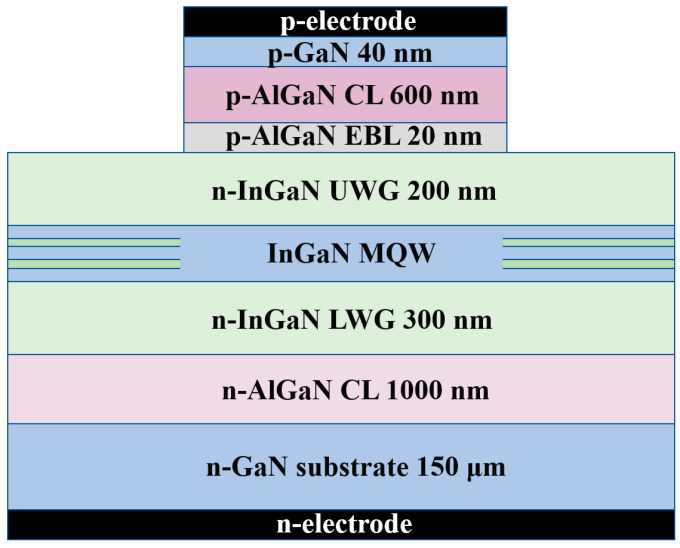
Schematic diagram of InGaN-based green LD structure for simulation by LASTIP.

A conventional ridge waveguide green LD structure is used as a reference in this work, and the schematic diagram of this green LD structure is shown in Figure 1. It consists of a 150 μm thick n-GaN substrate (Si doping = 3 × 10^18^ cm^−3^), a 1 μm thick n-Al_0.08_ Ga_0.92_ N cladding layer (n-CL) (Si doping = 3 × 10^18^ cm^−3^), a 300 nm thick n-In_0.08_ Ga_0.92_ N lower waveguide layer (LWG) (Si doping = 1 × 10^17^ cm^−3^), an InGaN multi-quantum well (MQW) active region designed for a target lasing wavelength of 520 nm, which consists of two 2.5 nm thick In_0.23_ Ga_0.77_ N quantum well layers sandwiched by three 10 nm thick In_0.02_ Ga_0.98_ N quantum barrier layers, a 200 nm thick n-In_0.04_ Ga_0.96_ N upper waveguide layer (UWG) (Si doping = 1 × 10^17^ cm^−3^), a 20 nm thick p-Al_0.15_ Ga_0.85_ N electron blocking layer (EBL) (Mg doping = 1 × 10^19^ cm^−3^), a 600 nm thick p-Al_0.08_ Ga_0.92_ N cladding layer (p-CL) (Mg doping = 2 × 10^19^ cm^−3^), and a 40 nm thick p-GaN contact layer (Mg doping = 1 × 10^20^ cm^−3^). The p-electrode and n-electrode are on the top side and bottom side of the LD structure. The ridge width and cavity lengths are 15 μm and 150 μm, respectively. During simulation, an ideal ohmic contact is set to both the n-electrode and p-electrode. Additionally, the refractive indices and band gaps of GaN-based materials are obtained according to previous works [17,18].

Based on the conventional green LDs, two sets of different green LDs are designed in order to optimize carrier injection efficiency. In series I, seven LDs with varied indium content from 1% to 7% in quantum barrier layers are studied. In series II, a set of LDs with a new composite LWG structure is proposed and the effect is calculated. The composite LWG structure consists of an In_0.04_ Ga_0.96_ N inserting layer and an In_0.08_ Ga_0.92_ N layer. The total thickness of composite LWG layer is kept to 300 nm, while the thickness of In_0.04_ Ga_0.96_ N inserting layer varies from 50 nm, 20 nm, 5 nm, 3 nm, and 1 nm, respectively. The lasing wavelength of all these samples is still 520 nm under the condition of a fixed 23% In composition of the well layer, as shown by the results of simulation.

## 3. Results and Discussion

### 3.1. Improvement of Output Characteristics by Modulating Indium Content in Quantum Barriers

**Figure 2:**
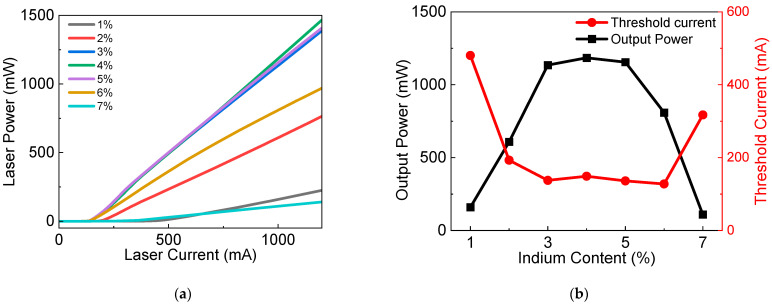
(**a**) Output laser power versus injected current (P-I), and (**b**) output power and threshold current versus indium content in quantum barriers. (All the output power data are collected under the same injection current of 1000 mA).

P-I curves of seven LDs in series I are displayed in Figure 2a, and a remarkable influence of In content in InGaN barrier layers on P-I curves can be observed. The threshold current and output power at a laser current of 1000 mA is obtained from P-I curves as summarized in Figure 2b. An interesting result is found that the threshold current of LDs decreases first and then increases with the increasing indium content of quantum barriers, and the output power of LDs increases first and then decreases, respectively. The performance of LD is optimized when the indium content of quantum barriers is set between 3% and 5%.

**Figure 3:**
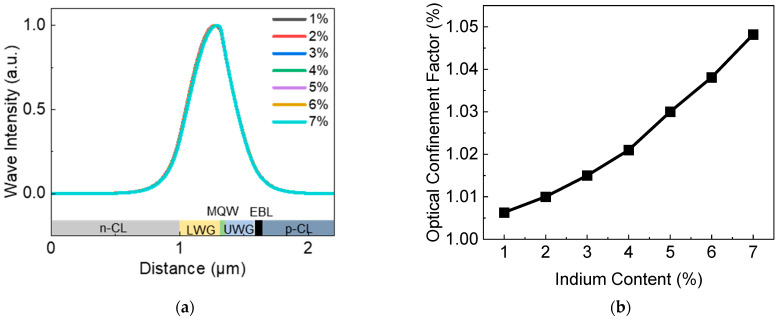
(**a**) Optical field distribution and (**b**) optical confinement factors versus indium content of quantum barriers.

The influence of indium content of quantum barriers on the optical properties of LDs is investigated first. It was thought that the optical field distribution might affect internal optical absorption loss significantly [19]. Practically, in order to minimize the optical loss in the P-type region, the effective index contrast between InGaN LWG and AlGaN n-cladding layer should be relatively larger, and a high indium content LWG and a rather low indium content UWG are used in green LDs [20]. Such an asymmetrical LWG and UWG design can help to realize asymmetrical optical field distribution [10]. As shown in Figure 3, however, only a very small change in optical field distribution and optical confinement factor can be observed, which has a nearly negligible influence on the simulated result. Additionally, Figure 3a also indicates that all these InGaN/InGaN MQW device structures have sufficiently good optical confinement within the waveguide region, and the optical field extends more to the n-side instead of to the p-side. Thus, the optical loss of LDs can be reduced effectively.

**Figure 4:**
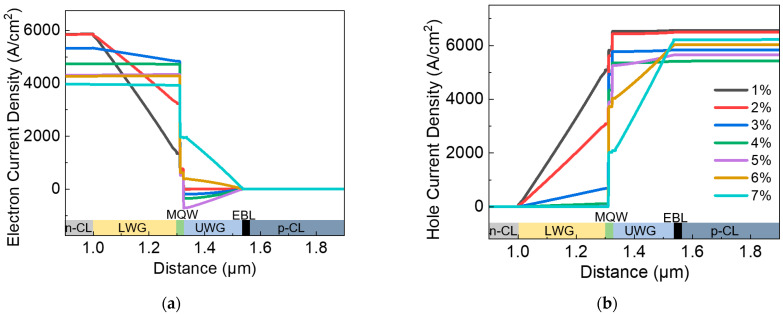
(**a**) Electron current density distribution and (**b**) hole current density distribution at the middle axis of device under the same injection current of 1000 mA.

Figure 4 shows the simulation results of vertical variations of injected electrons and holes around the MQW region of the LD structures. All the current density (A/cm^2^) data are collected under the same injection current of 1000 mA. Because of the 2D distribution of current density inside the device, current densities at the middle axis of the device are captured for comparison in this part. As shown in Figure 4a, a large decrease in vertical electron current density occurs in InGaN LWG when the indium content of the quantum barrier is 2% or below, which indicates that there is a strong recombination of injected electrons in the LWG layer. In Figure 4b, a strong recombination of injected holes occurs in UWG layers when the indium content of quantum barriers is above 5%. In other words, the unwanted extra recombination of injected carriers in the waveguide layers becomes reduced when the indium content of quantum barriers is around 3~5%, resulting in high injection efficiency and LD performance, which is in good agreement with the results obtained in Figure 2b.

**Figure 5:**
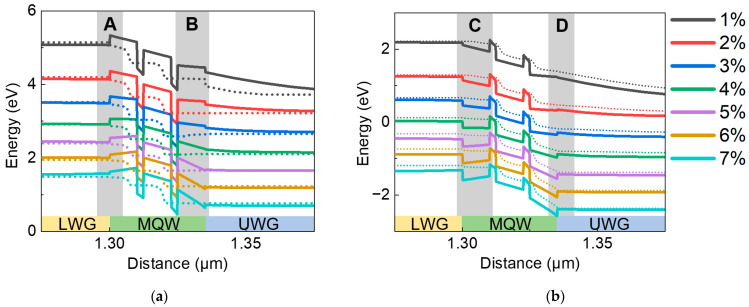
Conduction (**a**) and valence (**b**) band diagrams with different indium content of quantum barriers in the MQW region at an injection current of 1000 mA. In these figures, A, B, C, D represent potential barrier heights for electron injection, electron leakage, hole leakage and hole injection, respectively. (Solid lines represent conduction and valence bands, and dashed lines represent the position of quasi-Fermi level. The band curves of samples with different indium content are vertically shifted by 0.5 eV in sequence for easy eye view).

To further investigate the reason for LD performance differences, band structures of LDs are studied. The simulated result of the band diagram of LDs with various indium content in quantum barriers is shown in Figure 5a,b. An obvious change in potential barrier height, at both the p-side and n-side of the MQW active region, can be observed. Potential barriers for electron injection are marked as shaded area A and for the electron leakage are marked as shaded area B in Figure 5a. Potential barriers for hole leakage are marked as shaded area C, and for the hole injection are marked as shaded area D in Figure 5b. With increasing indium content in quantum barriers, the potential barrier height for electron injection from LWG to the quantum well region decreases (Figure 5a), which may have both positive and negative effects on the performance of the LDs. It results in an increasing electron injection efficiency, but at the same time, the electron leakage through MQW will also become easier. On the other hand, when indium content increases in quantum barriers, the potential barrier height for hole injection from UWG to the quantum well region will increase, and more holes will be blocked in UWG (Figure 5b), resulting in lower hole injection efficiency, and the hole leakage through the MQW may become reduced. Actually, since green LDs usually operate at high current density [7], severe negative influence may occur due to the carrier injection blockage effect even if carrier leakage is also not negligible in this case.

### 3.2. Output Characteristics Improvement by Using Composite LWG Layer

**Figure 6:**
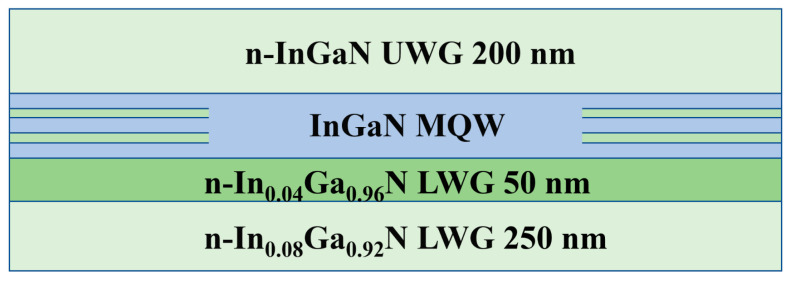
Schematical diagram for studied MQW and waveguide regions of a new LD structure with composite lower waveguide structure.

It is noted that the high potential barrier between the waveguide layer and active region may result in deterioration of injection efficiency of green LDs. In order to reduce the negative effect of the high potential barrier between the LWG and active region, a new composite LWG structure is proposed. Based on the conventional green LD structure which is with 2% indium content quantum barrier, five LDs with composite LWG layers are designed, where the 300 nm thick In_0.08_ Ga_0.92_ N LWG layer is replaced with a composite waveguide layer. The LWG of the first LD consists of a 250 nm thick In_0.08_ Ga_0.92_ N layer and a 50 nm thick In_0.04_ Ga_0.96_ N inserting layer as schematically shown in Figure 6. Additionally, one of the other four LD structures has a composite LWG structure consisting of 280 nm, 295 nm, 297 nm, 299 nm thick In_0.08_ Ga_0.92_ N layers and 20 nm, 5 nm, 3 nm, 1 nm thick In_0.04_ Ga_0.96_ N layers, respectively.

**Figure 7:**
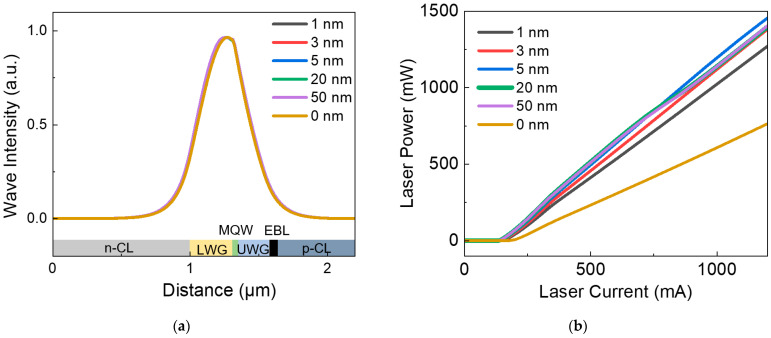
(**a**) Optical field distribution and (**b**) output laser power versus injected current (P-I) with different thickness of In_0.04_ Ga_0.96_ N inserting layer in the LWG.

The optical characteristics for conventional LD (marked as 0 nm) and five LDs with composite waveguide structures (the extra inserting In_0.04_ Ga_0.96_ N layer of 1, 3, 5, 20, 50 nm) are studied first. The simulation results of optical field distribution are shown in Figure 7a. It is found that different thicknesses of the InGaN inserting layer has little effect on optical field distribution. Thus, optical loss change can be neglected in the discussion on output efficiency change. The LD P-I curves with different inserting layer thicknesses are shown in Figure 7b. A remarkable improvement in output power at 1000 mA and a reduced threshold current for composite waveguide structures can be observed comparing to conventional single-layer LWG structures. However, the thickness of the inserting layer does not show any significant influence on output power and threshold current of LDs when the thickness is over 5 nm. When the thickness of the inserting LWG layer decreases to 1 nm, a deterioration in output efficiency can be observed. The best choice for the inserting LWG layer thickness is around 5 nm, with the highest slope efficiency. Simulations with thicknesses of inserting layers under 1 nm are of small reference significance, as a 1 nm thick inserting layer has only three atomic monolayers in the real crystalline structure.

**Figure 8:**
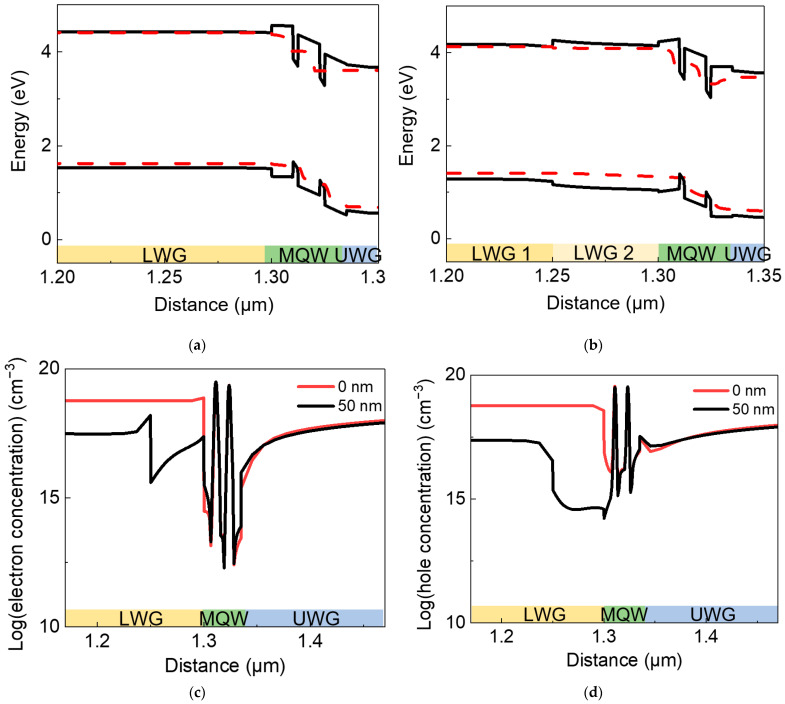
Energy band diagrams of (**a**) conventional single layer LWG structure and (**b**) composite structure with a 50 nm thick In_0.04_ Ga_0.96_ N inserting layer at an injection current of 1000 mA, where the black solid lines represent conduction and valence bands, and red dashed lines represent quasi-Ferimi level. (**c**) Electron concentration and (**d**) hole concentration distribution in two LDs with different LWG structures with or without an inserting layer at an injection current of 1000 mA.

For further investigation, band structures of LDs with conventional LWG structure and composite LWG structure consisting of 50 nm-thick inserting layer are presented as shown in Figure 8a,b. A high effective electron potential barrier between LWG layer and MQW active region in Figure 8a is divided into two smaller potential barriers in the LWG in Figure 8b, which reduces the difficulty for electron injection. Moreover, a higher and wider hole potential barrier between LWG and MQW is observed in Figure 8b, remarkably reducing hole leakage. As shown in Figure 8c, the new structure decreases electron concentration in LWG layer significantly. More importantly, hole leakage is prevented effectively as a sharp decrease in hole concentration in the inserted layer can be seen in Figure 8d, and the hole concentration in the In0.08 Ga0.92 N LWG layer is also reduced by about two orders of magnitude. Thus, the total unwanted extra recombination in the LWG layer decreases, and the injection efficiency is improved, resulting in a threshold current decrease and slope efficiency increase in the green LD. In general, the composite LWG structure is proved to be an effective method to enhance the electron injection and reduce the current leakage, improving the performances of InGaN-based green LDs.

## 4. Conclusions

In conclusion, a systematical investigation of the effects of potential barriers between waveguide layers and the MQW active region in InGaN/InGaN-based green LDs is carried out by LASTIP simulation. It is found that LDs with higher indium content quantum barriers have stronger recombination in the UWG layer, and LDs with lower indium content quantum barriers have stronger recombination in the LWG layer. An appropriate indium content of quantum barriers, around 3% to 5%, can remarkably reduce the total recombination in the LWG and UWG layers. Additionally, a new structure with a composite InGaN LWG layer consisting of In_0.04_ Ga_0.96_ N inserting layer and In_0.08_ Ga_0.92_ N layer is proposed to reduce the negative effects of high potential barriers induced by the low indium content LWG layer. Additionally, this new structure is demonstrated to be effective in improving the output characteristics of green LDs owing to its advantages of reducing electron blocking effects and hole leakage.

## Data Availability

The data that support the findings of this study are available from the corresponding author upon reasonable request.
